# The Role of Proton Therapy for Prostate Cancer in the Setting of Hip Prosthesis

**DOI:** 10.3390/cancers16020330

**Published:** 2024-01-11

**Authors:** Maryam Moteabbed, Mislav Bobić, Harald Paganetti, Jason A. Efstathiou

**Affiliations:** 1 Department of Radiation Oncology, Massachusetts General Hospital and Harvard Medical School, Boston, MA 02114, USAjefstathiou@partners.org (J.A.E.); 2 Department of Physics, ETH Zurich, 8093 Zurich, Switzerland

**Keywords:** prostate cancer, hip prosthesis, proton arc therapy

## Abstract

**Simple Summary:**

As proton therapy evolves and becomes more accessible, it is important to explore its potential value for all patient cases to enable more informed treatment individualization. Currently, external beam radiotherapy for prostate cancer patients with hip prosthesis is limited to photons because traditional proton therapy beams interfere with the implanted hardware. The goal of this study was to investigate the dosimetric feasibility and robustness of novel proton therapy techniques, i.e., proton arc therapy and anterior beam directions for prostate cancer patients with unilateral hip prosthesis. It was demonstrated that proton therapy for prostate cancer in the setting of hip prosthesis is feasible and could potentially reduce the dose to some critical organs and hence reduce toxicities.

**Abstract:**

Purpose: Given that the current standard of proton therapy (PT) for prostate cancer is through bilateral beams, this modality is typically avoided when it comes to treatment of patients with hip prosthesis. The purpose of this study was to evaluate whether novel PT methods, i.e., anterior proton beams and proton arc therapy (PArc), could be feasible options to treat this patient subpopulation. We evaluate PT methods in the context of dosimetry and robustness and compare with standard of practice volumetric modulated arc therapy (VMAT) to explore any potential benefits. Methods: Two PT and one VMAT treatment plans were retrospectively created for 10 patients who participated in a clinical trial with a weekly repeat CT (rCT) imaging component. All plans were robustly optimized and featured: (1) combination anterior oblique and lateral proton beams (AoL), (2) PArc, and (3) VMAT. All patients had hydrogel spacers in place, which enabled safe application of anterior proton beams. The planned dose was 70 Gy (RBE) to the entire prostate gland and 50 Gy (RBE) to the proximal seminal vesicles in 28 fractions. Along with plan dose–volume metrics, robustness to setup and interfractional variations were evaluated using the weekly rCT images. The linear energy transfer (LET)-weighted dose was evaluated for PArc plans to ensure urethra sparing given the typical high-LET region at the end of range. Results: Both PT methods were dosimetrically feasible and provided reduction of some key OAR metrics compared to VMAT except for penile bulb, while providing equally good target coverage. Significant differences in median rectum V35 (22–25%), penile bulb Dmean (5 Gy), rectum V61 (2%), right femoral head Dmean (5 Gy), and bladder V39 (4%) were found between PT and VMAT. All plans were equally robust to variations. LET-weighted dose in urethra was equivalent to the physical dose for PArc plans and hence no added urethral toxicity was expected. Conclusions: PT for treatment of prostate cancer patients with hip prosthesis is feasible and equivalent or potentially superior to VMAT in quality in some cases. The choice of radiotherapy regimen can be personalized based on patient characteristics to achieve the best treatment outcome.

## 1. Introduction

Nearly 2 million cases of new cancers and 609,820 cancer deaths are projected in the United States in 2023, out of which almost 288,000 men are estimated to be diagnosed with prostate cancer. Prostate cancer is one of the most common types of cancer, with its incidence increasing with age. The average age at the time of diagnosis is 66 years. It is generally characterized by prostate enlargement, problems with urination and rising prostate-specific antigen (PSA). It is mostly slow growing and localized at the time of diagnosis. Many patients with early stage prostate cancer do not receive any treatment and opt for active surveillance. However, some types of prostate cancer could be more aggressive and require treatment, including surgery, radiation or radiopharmaceutical therapy, hormone therapy, chemotherapy, targeted therapy, and immunotherapy. While surgery and radiation therapy (RT) are primary options for localized cancer with comparable outcomes, systemic therapies, e.g., chemotherapy, are typically used in concurrent or adjuvant settings and for higher-stage disease, and typically have more side effects [1,2].

**Figure 1:**
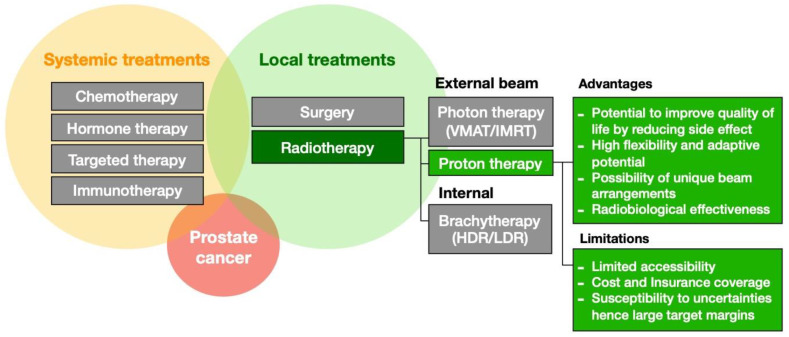
Treatment options for prostate cancer beyond active surveillance. The role of proton therapy and its advantages and limitations are highlighted in green.

More than 25% of patients with prostate cancer will receive RT. It is used as a primary option for localized disease and as part of the treatment regimen for advanced or recurrent disease. RT could be delivered via external beams, e.g., photon and protons, or internally through brachytherapy [1,2]. Particle therapy, most commonly proton therapy (PT), has been shown to reduce the low-dose bath to the nearby tissues significantly compared with photon-based RT, thus effectively reducing the toxicities for different disease sites [3,4,5,6]. For prostate cancer, PT is not a standard care and common treatment option due to unclear clinical advantages over the existing methods to justify its higher cost. Randomized controlled clinical trials are currently investigating the efficacy of PT compared to conventional photon-based RT [7]. Figure 1 illustrates the treatment option or prostate cancer and lists the advantages and limitations of PT.

However, PT has been evolving toward faster and more precise delivery methods. Most notable of these advances are the pencil beam scanning (PBS) technique, enabling intensity-modulated dose painting [8]; image-guided adaptive techniques, allowing for target margin reduction and hypofractionation [9]; radio-biologically driven optimization to exploit particle beams’ enhanced radiobiological effectiveness [10]; and other emerging investigational techniques, e.g., proton arc therapy (PArc), delivering continuous proton beam during a gantry rotation [11], and FLASH proton therapy, delivering doses at very high dose rates [12]. Although PT for prostate cancer still mainly follows the standard guidelines, i.e., bilateral opposed beams that directly cross the femoral heads and deliver uniform dose to prostate, more sophisticated treatment methods can be explored, especially for nonstandard patient cases.

The prevalence of total hip replacement in the U.S. is also strongly correlated with age. It is mainly due to osteoarthritis and has been shown to increase from 0.6% to 5.9% from 50 to 90 years of age, with an average age to undergo the procedure of 67.2 years [13]. Therefore, a substantial number of prostate cancer patients who benefit from RT are expected to have some extent of hip prosthesis. The high density of the prosthesis material, most commonly titanium (density = 4.5 g/cm^3^), leads to added complexity and uncertainties in RT treatment planning. This is due to beam attenuation and scatter, the severity of which could depend on the beam energy and prosthesis material. Therefore, beam arrangements that avoid the prosthesis region have been recommended to decrease uncertainties [14]. The current standard of RT practice for prostate cancer patients with hip prosthesis is volumetric modulated arc therapy (VMAT), which delivers the prescribed dose during a continuous rotation of the gantry around the isocenter, using prosthesis avoidance techniques, e.g., several partial arcs that could be cumbersome, and applying either avoidance sectors or avoidance optimization with full arcs [15,16,17,18,19,20]. PT has not been a feasible option so far due to beam interference with the prosthesis hardware. Therefore, patients with hip replacements are typically excluded from randomized clinical trials with a PT arm.

In light of recent advances in PT technology and in hopes of reducing the burden of toxicities and improving quality of life for all patients equally, the role of proton therapy for the patient subpopulation with hip prosthesis needs to be further clarified. Notably, PArc has demonstrated great potential to improve treatment quality for prostate cancer over bilateral robustly optimized proton pencil beam scanning, while being deliverable within a clinically acceptable time [21]. Specifically, PArc could significantly spare the femoral heads compared to the conventional PT method [21,22]. Although PArc is a new modality, it is gaining increased interest and is an active area of research and the clinical implementation is ongoing [11,23,24,25]. Another solution to achieving femoral head sparing in prostate PT is through alternative beam angles [26,27,28]. Although anterior oblique beams can minimize femoral head dose at the expense of slight increase in the bladder dose compared with lateral beams, they have previously been associated with reduced robustness to interfractional variations that could lead to target underdose in the absence of adaptive range verification or robust optimization [29]. With the recent replacement of endorectal balloons by hydrogel spacers, the application of robust optimization for anterior beams is now safe and feasible, maintaining the plan integrity against interfractional variations [30].

In this study, we aim to explore whether PT could be considered as a feasible effective and even potentially superior treatment option for prostate cancer patients with unilateral hip prosthesis by offering equal or better treatment quality to VMAT. We compare the dosimetric quality and robustness of PArc and anterior proton beams to VMAT and further explore the feasibility of PArc for prostate cancer treatments by assessing possible elevation of biological effectiveness in the central prostate region and its implications on urethral dose and potentially toxicity.

## 2. Methods and Materials

### 2.1. Patient Characteristics

Ten patients with low- and intermediate-risk prostate cancer participating in a randomized multicenter clinical trial who consented to undergo an optional weekly repeat CT (rCT) imaging at the Massachusetts General Hospital were included in this study [7]. The simulation CT was used for treatment planning and the 6 rCT scans were used to evaluate treatment robustness to setup and interfractional variations. rCTs were acquired on the same single energy CT scanner as the simulation image (GE Healthcare, Boston, MA, USA) using the same setup protocols and parameters. Patients were encouraged to keep the bladder and rectal filling consistent with the simulation and treatment sessions by drinking water and emptying their bowels prior to the scans. Patients had 3 gold fiducial markers implanted within their prostate gland for setup guidance and biodegradable hydrogel spacers injected to create temporary space between prostate and rectum for enhanced rectal sparing and immobilization. MR-guided intervention was used for spacer placement. No endorectal balloons were used. Because patients with hip prosthesis were not eligible to participate in the randomized portion of the clinical trial from which the imaging data were collected, the prosthesis was simulated by overriding the density of the left femoral head to the density of titanium (4.5 g/cm^3^).

### 2.2. Treatment Planning and Robustness Evaluation

Three new treatment plans were created for each patient using RayStation V10B treatment planning system (RaySearch Laboratories, Stockholm, Sweden): Two pencil beam scanning PT plans included a 3-field proton plan with 2 anterior oblique beams and a lateral beam through the healthy (right) hip. The second was a PArc plan, created by placing 16 beams separated by 20-degree increments from 190 to 350 degrees, at 0- and 180-degree couch rotations (PROTOM Radiance 330 Synchrotron System). No beams were placed around the left hip region as an avoidance strategy. The incremental static beams were used because no PArc planning or optimization strategy was available in the RayStation package being used at the time. Conventional optimization was applied with robustness criteria including 4 mm setup and 3% range uncertainties. The third plan was VMAT using 2 full arcs (clockwise and counterclockwise). The prosthesis region was avoided by applying a strict optimization constraint (<500 cGy to 10% volume) [15,31]. Dose to the prosthesis volume was assessed to evaluate the avoidance efficiency, because this criterion was not prioritized over target coverage during optimization. Target volumes were defined as CTV70 as the whole prostate and CTV50 as the prostate plus proximal seminal vesicles. The dose for all planning methods was 50 Gy to CTV50 plus an additional 20 Gy to CTV70 for a total of 70 Gy(RBE) in 28 fractions. All planning constraints were adapted from the clinical trial protocol [7] (See Table 1).

The delivered dose when accounting for anatomy and setup variations was derived as follows: First, external surface as well as bladder and rectum were contoured on each rCT. RayStation deformable image registration module was used to deform each fused rCT to the simCT using bladder and rectum as controlling structures to yield more accuracy in the presence of large organ filling variations [32]. The planned dose for each case was deformed according to each simCT–rCT deformation vector field and applied to 3–5 fractions (1 week of treatment). The six deformed doses (per patient per plan) were then accumulated to derive the total estimated delivered dose after accounting for setup and anatomy variations. The accumulated doses were exported using the Python scripting module within RayStation for further analysis.

### 2.3. LET Modeling

The proton end of range is associated with increased relative biological effectiveness (RBE) [33]. LET-weighted dose calculation is a technique to reduce biological variability in PT planning [10]. Because in PArc plans, the proton end of range is expected to fall close to the center of prostate, where the urethra is typically located, we further investigated the LET-weighted dose in the urethra (𝛋 = 0.052) to evaluate clinical acceptability of conventionally optimized PArc. The urethra structure was additionally delineated on the T2 MRI images available for all patients as a result of spacer placement [34,35]. This structure was then propagated onto simCT through deformable registration.

We used the GPU-based Monte Carlo (MC) package MOQUI [36] to calculate LET-weighted dose using the PArc plan parameters, e.g., plan name, external structure name, and Hounsfield Unit override information. MOQUI is a novel code capable of scoring large quantities of particles efficiently and accurately by integrating parallel particle transportation with a hash table key value pair data structure.

### 2.4. Data Analysis

Dose–volume histograms (DVH) were exported from each plan and analyzed using R (R software v 4.3.2 for statistical computing) [37]. The dose volume metrics analyzed include dose at 95 and 5% volume (D95 and D5) for the target volumes, mean dose (Dmean) and volumes at 66 and 35 Gy dose (V66, V35) for bladder and rectum, Dmean for the penile bulb, V39 for the right femoral head, and V5 for the prosthesis. The differences between delivered and planned parameters for each key structure were used as an indicator of plan robustness.

Furthermore, clinical outcomes were compared by using Lyman Kutcher Burman (LKB) normal tissue complication probability (NTCP) modeling. Key endpoints, i.e., grade 2 rectal bleeding, grade ≥ 1 and ≥2 urinary toxicity including urinary frequency, urgency, incontinence and cystitis, and grade ≥3 hematologic toxicity, were evaluated. Model parameters used included m = 0.16, n = 0.1, TD50 = 73.6 Gy for rectum [38], and m = 0.43, n = 0.19, TD50 = 59.5 Gy/m = 0.27, n = 0.14, TD50 = 81.9 Gy for bladder G1+/G2+, respectively [39], and m = 0.27, n = 1.0, TD50 = 35 Gy for the right femoral head bone marrow [40].

The DVH for the LET-weighted dose distribution was also analyzed and compared with the physical dose distribution to assess the potential impact of LET on the urethral exposure and risk of toxicity.

Wilcoxon signed-rank statistical test was performed to assess the significance of the differences in dose–volume metrics and robustness (*p* < 0.05 was considered significant). The statistical analysis was performed using R software.

## 3. Results

### 3.1. Dosimetric and Outcome Comparison

**Figure 2:**
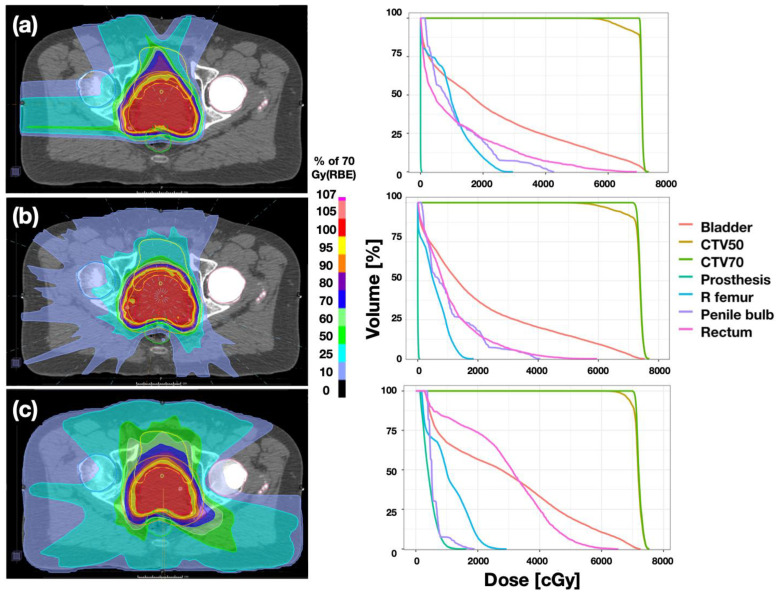
Dose distributions featuring (**a**) AoL, (**b**) PArc, and (**c**) VMAT plans, for a representative patient. The respective DVHs are shown on the right panel.

The dose distributions are compared among AoL, PArc, and VMAT plans in Figure 2 for a representative patient. DVHs are also shown for each respective case. The right femoral head density was overridden to represent a titanium hip prosthesis as illustrated. The initial visual inspection indicates that the main difference between the three plans is the distribution of low and intermediate dose throughout the patient anatomy, as target coverage is comparable among plans. Note that the prescribed dose volume is larger in the PT plans because the robust optimization criteria also includes range uncertainty unlike VMAT plans, which only include setup uncertainties. DVHs show lowest OAR doses in PArc plans, except the penile bulb dose, which was lowest in VMAT plans for all cases. Both proton plans delivered negligible dose to the prosthesis volume whereas the prosthesis dose in VMAT could not be reduced any further than the tolerance set for optimization due to target coverage loss.

**Figure 3:**
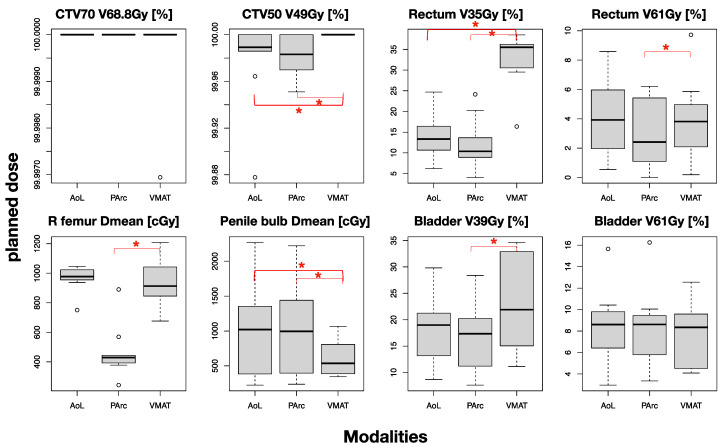
Box–whisker plots showing the distribution of the key DVH metrics over all patients for targets and OARs, comparing the three planning modalities. Red asterisks indicate significant difference between proton and VMAT plans.

Figure 3 demonstrates box plots comparing dose volume metrics among plans. The significance of the difference between each proton plan and the VMAT plan is illustrated by red asterisks. Significant reduction in rectum V35 (22–25%) and penile bulb Dmean (5 Gy) were found for both PT plans compared to VMAT, whereas significant reduction in rectum V61 (2%), right femoral head Dmean (5 Gy), and bladder V39 (4%) were observed between PArc only and VMAT. The significant difference in CTV50 coverage was not clinically relevant since all values were well above the prescribed dose. The femoral head V39 for all plans was 0; therefore, Dmean was assessed. Note, however, that all plans satisfy all the clinical constraints (see Table 1).

**Figure 4:**
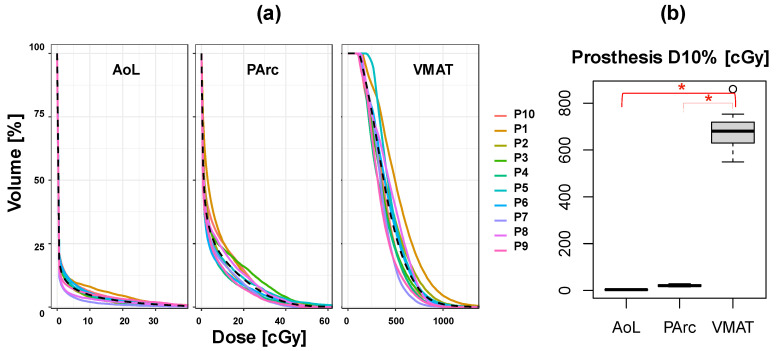
(**a**) DVH comparison between planning modalities for the prosthesis volume for all patients. (**b**) Box plot comparing prosthesis D10 among plans for each planning modality. Red asterisks indicate significant difference.

Figure 4 shows the DVH for the prosthesis volume for all patients and all plans. The median prosthesis D10 is almost zero for both PT plans, whereas it is approximately 650 cGy for the VMAT plans and significantly larger than PT.

Radiobiological analysis of the DVH distributions showed no significant difference between modeled rectal and urinary toxicities among the three planning methods. The median NTCP for G2 rectal bleeding for AoL, PArc, and VMAT were 2.16, 1.29, and 1.84%, respectively. Median G1+/G2+ urinary toxicities for AoL, PArc, and VMAT were 29.15/7.55%, 28.8/7.4%, and 28.7/7.15%, respectively. The G3 hematologic toxicity for the healthy femoral head bone marrow was similar between AoL and VMAT but 5–6 times smaller for PArc.

### 3.2. Robustness Comparison

**Figure 5:**
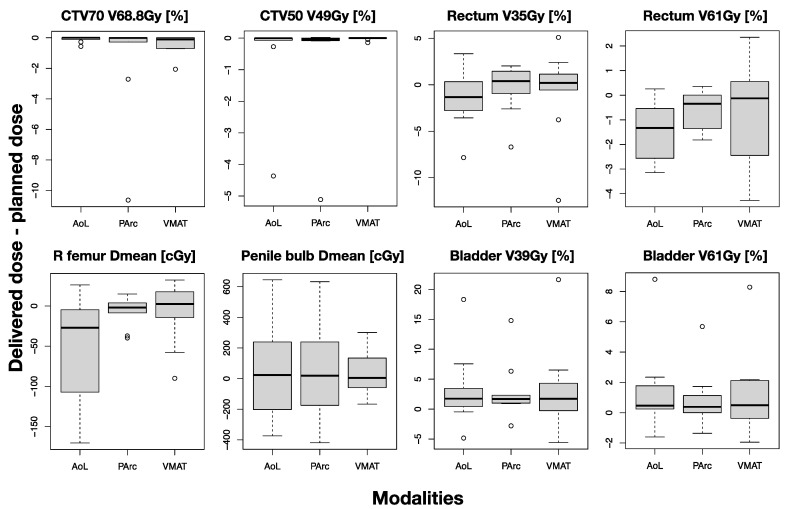
Box–whisker plots showing the distribution of the differences between delivered and planned DVH metrics over all patients for targets and OARs, comparing the three planning modalities. No significant differences were detected.

The delivered dose differed from the planned dose due to setup and anatomy variations. Figure 5 shows the difference between delivered and planned dose metrics for the targets and relevant OARs for all patients. In most cases the variations stayed within the clinical constraints. Minor deviations occurred for bladder V61 on all plans (patient 1), bladder V39 on VMAT (patient 1) and rectum V35 (patient 2), and CTV70 V68.8 on VMAT (patient 2) and PArc (patients 7 and 9). No significant differences were found between the robustness of proton techniques and VMAT for any of the metrics.

### 3.3. LET-Weighted Dose to Urethra for PArc

**Figure 6:**
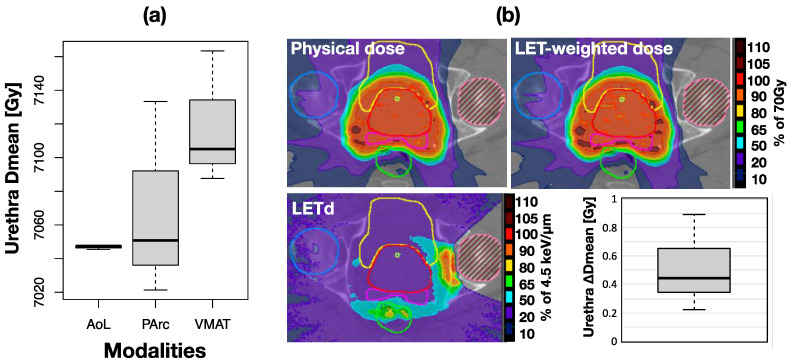
(**a**) Box plot comparison of urethral physical Dmean between the planning modalities. (**b**) Distributions of physical and LET-weighted dose, and LETd for a representative case. The box plot represents the difference between urethra LET-weighted and physical Dmean for all patients.

The planned median urethra Dmean among PArc plans was slightly (0.5 Gy) smaller for the proton than photon plans as illustrated in Figure 6a. Figure 6b shows the distributions of the physical dose, LET-weighted dose, and the dose-averaged LET (LETd) for a representative case. The regions of relative LET elevation were located in the periphery of the prostate and outside of the prosthesis volume, as well as close to the rectum. However, the peak LETd was only approximately 5 keV/μm. The LETd within the prostate was on average 1.85 keV/μm (1.7–2.0 keV/μm). The median of the difference between LET-weighted dose and physical dose was 0.45Gy as illustrated in the box plot also in Figure 6b. The effect of the slight increase in LET on the rectum dose was found to be negligible. The rectum Dmean difference between LET-weighted and physical doses was within 0.6 Gy for all patients.

## 4. Discussion

While pursuing the goal of cancer treatment individualization for all patients regardless of their special circumstances, it is important to actively explore the role of novel treatment options made available by current technological advancements. We aimed to study the role of proton therapy as a possible treatment option for prostate cancer patients with hip prosthesis and investigate any potential benefits PT could offer to this patient cohort that would exceed the current standard of care, i.e., VMAT. We studied PT delivery by hybrid anterior and lateral oblique beams and proton arc therapy and compared the dosimetric characteristics and robustness of each method with VMAT. Ten patients with weekly repeat CT scans were used and three robustly optimized plans (two PT and a VMAT) were created and recalculated on repeat CTs to take the anatomy variations into account. We found that both proton plans offered the opportunity to fully avoid the prosthesis volume, which was simulated by overriding the density of the left femoral heads. PArc plans additionally significantly reduced several dose–volume metrics for the right femoral head, bladder, and rectum compared to VMAT, while VMAT significantly reduced the penile bulb mean dose compared to both proton methods. In case of VMAT optimization, a strong tradeoff existed between reducing the prosthesis dose and maintaining the target coverage. Therefore, the prosthesis dose could not be minimized to the same degree as the PT plans. No significant difference was found between the robustness of the modalities to interfractional variations neither for target coverage nor OAR dose. NTCPs with endpoints of grade 2+ rectal and urinary toxicities were comparable between modalities and with the endpoint of hematologic toxicities due to femoral head bone marrow irradiation being significantly lower for PArc. Furthermore, PArc did not increase the urethral biological dose as a result of concentric beams and the end of range LET peak. The LET-weighted dose was only slightly more elevated than the physical dose around the periphery of the prostate and did not negatively impact any OARs.

No previous studies have investigated the efficacy of different PT techniques for treatment of prostate cancer patients with hip prosthesis compared with VMAT. A few clinical studies have evaluated the safety of hip-avoiding PT for unilateral hip prosthesis and found that no additional urinary, rectal, or skeletal complications were reported by the five patients included [27,41]. Rana et al. dosimetrically compared hybrid lateral oblique uniform scanning proton beams to VMAT and found potential dosimetric advantages [26]. We previously studied the robustness of anterior proton beams to interfractional variations and concluded that target coverage can be severely compromised if range variations are not fully compensated throughout treatment fractions [29]. In this study, patients had been injected with hydrogel spacers prior to treatment, which therefore provided an appropriate setting for using robust optimization to create anterior proton beam plans with high quality and integrity. We also explored the dosimetry and robustness of PArc in the context of hip prosthesis for the first time. Even though PArc is not yet clinically available, it is in prototyping phase and expected to emerge in the clinics in the near future [11].

A potential limitation of this study could be the lack of actual hip prosthetic hardware in the patient cohort. The reason for choosing these patients is twofold: First, the data used were taken from a randomized clinical trial with a weekly imaging component, which allowed for collecting repeat CTs for robustness analysis. Furthermore, patients also had implanted hydrogel spacers, which was an important aspect of hip-avoiding proton therapy planning. Second, patients with hip replacements are not extremely common; hence, the data collection time would have been extended considerably. The density override applied to simulate the prosthesis did not include the streaking metal artifacts in the CT images typically seen in the presence of such hardware. Hence, the impact of metal artifacts is not included in the current report. However, it is expected that this impact would be negligible because both proton plans fully avoided the prosthetic hip and its close proximity and given the current sophisticated metal artifact reduction software on most CT systems, e.g., Smart Metal Artifact Reduction (MAR) by GE Healthcare or Philip’s Orthopedic Metal Artifact Reduction (OMAR) [42,43]. Any remaining artifacts missed by these algorithms can be manually corrected by density overrides, so the lack of artifacts does not imply an inaccuracy in dose. An additional limitation of this study could be the relatively small sample size obtained from a single institution. Given the exploratory nature of the research and considering the small patient variability, the findings serve as a valuable starting point to encourage larger future clinical investigations tailored specifically for this unique patient group.

It is worth noting that we only consider unilateral hip prosthesis in this study because it is relatively more common than bilateral hip replacement. We expect the findings to be similarly applicable to all prosthesis configurations. For these cases, the lateral proton beam in the AoL technique should be removed. Also worth mentioning is that conventional optimization (parameter tuning) was used in our study for both PT plans (for consistency) due to the unavailability of multicriteria arc optimization on the utilized version of the planning system. On the other hand, VMAT plans were created using multicriteria optimization (MCO). Although care was taken to optimize all plans using identical objective functions and constraints, some bias might still have been introduced, as MCO is known to improve treatment plan quality [44].

Although PT had advantages to VMAT in reducing the dose burden to several OARs, penile bulb dose was consistently and significantly lower for VMAT compared to proton plans, which could indicate clinical benefits of photons for patients with predisposition to erectile dysfunction. This emphasizes the importance of an individualized approach in radiotherapy of prostate cancer in the setting of hip prosthesis. PT, especially via arc delivery, is a great potential option for significantly reducing the dose to important OARs, offering a chance for improved quality of life and treatment outcomes for many patients. It is important to prioritize clinical availability and facilitate accessibility of such treatment method to patients who could significantly benefit from it.

## 5. Conclusions

PT can be a promising alternative to VMAT for prostate cancer patients with unilateral hip prosthesis. Anterior oblique proton beams and proton arc therapy are both safe and robust options, which not only minimize the dose to the prosthetic hip, thus minimizing dosimetric complexity, but also could further spare the contralateral femur, rectum, and bladder (especially PArc). Both PT methods are robust to interfractional anatomical and setup variations similarly to VMAT. PArc does not cause increased biological dose to the urethra and/or nearby OARs.

PT via anterior beams and PArc (when clinically available) could be considered a feasible option along with VMAT for the treatment of prostate cancer in the setting of hip prosthesis. The choice between the modalities could be personalized based on each patients’ characteristics, metabolic profiles, and known susceptibility to particular side effects. The findings highlight the importance of continued research and developments in PArc technology toward its clinical implementation. Future evidence-based clinical investigations can utilize this technology to optimize RT protocols for this patient subgroup.

Exploiting the advances in PT and novel treatment opportunities it offers can pave the way for individualization and inclusion in cancer treatment.

## Figures and Tables

**Table 1:** Relevant key dose volume tolerances used in this study.

	CTV70 V68.8 (%)	CTV50 V49 (%)	Bladder V39 (%)	Bladder V61 (%)	Rectum V35 (%)	Rectum V61 (%)	Femoral Head V39 (%)	Penile Bulb Dmean (Gy)
Tolerance	99	95	50	20	40	15	5	45

## Data Availability

The data are not publicly available due to privacy restrictions.

## Abbreviation

AoL	Anterior oblique lateral
LET	Linear energy transfer
LETd	Dose-averaged linear energy transfer
MCO	Multicriteria optimization
NTCP	Normal tissue complication probability
OAR	Organ at risk
PArc	Proton arc therapy
PSA	Prostate-specific antigen
PT	Proton therapy
RBE	Relative biological effectiveness
RT	Radiation therapy
VMAT	Volumetric modulated arc therapy
